# Higher incidence of pegfilgrastim-induced bone pain in younger patients receiving myelosuppressive chemotherapy: a real-world experience

**DOI:** 10.1186/s40780-022-00272-9

**Published:** 2023-01-10

**Authors:** Shinya Tsuboi, Tatsuya Hayama, Katsuhiro Miura, Akihiro Uchiike, Daisuke Tsutsumi, Takashi Yamauchi, Yoshihiro Hatta, Susumu Ootsuka

**Affiliations:** 1 Department of Pharmacy, Nihon University Itabashi Hospital, 30-1 Oyaguchikamicho, Itabashi City, Tokyo, 173-8610 Japan; 2 Tumor Center, Nihon University Itabashi Hospital, 30-1 Oyaguchikamicho, Itabashi City, Tokyo 173-8610 Japan; 3 Department of Medicine, Division of Hematology and Rheumatology, Nihon University School of Medicine, 30-1, Oyaguchikamicho, Itabashi City, Tokyo, 173-3610 Japan

**Keywords:** Age, Bone pain, Chemotherapy, Febrile neutropenia, Pegfilgrastim

## Abstract

**Background:**

Pegfilgrastim is widely used for the prevention of febrile neutropenia (FN) in patients receiving myelosuppressive chemotherapy for various types of cancer. However, pegfilgrastim-induced bone pain (PIBP) is a relevant adverse event occurring during cancer treatment. Thus, we aimed to determine the risk factors for PIBP in real-world clinical practice.

**Main body:**

We retrospectively collected the clinical records of patients who received pegfilgrastim to support myelosuppressive chemotherapy with at least a 10% risk of FN between 2015 and 2018 at our center. Patients received pegfilgrastim 3.6 mg between days 2 and 7 after chemotherapy administration (day 1) for primary or secondary prophylaxis against FN. All adverse events were recorded according to the Common Terminology Criteria for Adverse Events. Patients who experienced intermittent bone pain in the back, femur, or other anatomic sites after the pegfilgrastim administration were considered to have PIBP. To evaluate the relationship between PIBP incidence and patient characteristics, we performed univariate and multivariate logistic regression analyses to calculate the odds ratios (ORs) of possible risk factors for PIBP. We analyzed the data of 305 patients (median age: 63 years), who underwent 1220 chemotherapy cycles with pegfilgrastim per cycle. Univariate analysis revealed that female sex (vs. male sex), younger age (< 55 years vs. ≥ 55 years), and solid cancers (vs. hematologic cancers) had significantly higher ORs (*p* < 0.05). However, only younger age (< 55 years) was an independent risk factor for PIBP on multivariate analysis (OR 3.62, 95% confidence interval 1.51–8.69, *p* = 0.004).

**Conclusions:**

Younger age (< 55 years) was significantly associated with a higher risk of PIBP among patients receiving chemotherapy with a ≥ 10% risk of FN. Therefore, oncologists should meticulously formulate management plan for PIBP in younger patients after administering pegfilgrastim.

## Background

Currently, granulocyte-colony stimulating factor (G-CSF) is widely used to prevent chemotherapy-induced myelosuppression and febrile neutropenia (FN) [1]. Pegfilgrastim, a PEGylated form of G-CSF with sustained effects on granulocyte progenitor cells, has shown similar efficacy as short-acting filgrastim. Compared to conventional G-CSFs requiring daily administration, pegfilgrastim only requires a single shot per chemotherapy cycle. Therefore, pegfilgrastim is currently preferred for patients requiring G-CSF support [2].

Bone pain is the most common adverse event associated with G-CSFs [3]. Bone pain resulting from G-CSF administration involves bone marrow expansion and inflammatory reactions [4]. The severity of bone pain induced by G-CSFs is usually mild or moderate; nonetheless, it results in deterioration of the patient’s quality of life.

An early randomized study reported that pegfilgrastim-induced bone pain (PIBP) occurred in 37% of patients, which did not significantly differ from the rate of bone pain induced by conventional filgrastim [5]. However, recent trials have indicated that the incidence rate of PIBP is 10–70% [6–9]. This discrepancy suggests that PIBP frequency can vary depending on the patient’s background characteristics and pegfilgrastim administration method, including dosage and timing. Although there has been significant interest from oncologists, risk factors for PIBP have not been established, since few studies have been conducted in real-world practice.

## Main text

This retrospective observational study used data from the Tumor Center of Nihon University Itabashi Hospital. Patients were considered eligible if they received intermediate- or high-risk chemotherapy for FN (i.e., ≥ 10% risk of FN) and primary or secondary pegfilgrastim prophylaxis at our center between August 2015 and May 2018. The chemotherapy regimens comprised epirubicin (90 mg/m^2^ intravenously on day 1, every 3 weeks) and cyclophosphamide (600 mg/m^2^ intravenously on day 1, every 3 weeks), docetaxel (75 mg/m^2^ intravenously on day 1, every 3 weeks) and cyclophosphamide (600 mg/m^2^ intravenously on day 1, every 3 weeks), docetaxel (75 mg/m^2^ intravenously on day 1, every 3 weeks) with or without trastuzumab (8 mg/kg intravenous loading dose, followed by 6 mg/kg every 3 weeks), docetaxel (75 mg/m^2^ intravenously on day 1, every 3 weeks) and trastuzumab (8 mg/kg intravenous loading dose, followed by 6 mg/kg every 3 weeks) and pertuzumab (840 mg/kg intravenous loading dose, followed by 420 mg/kg every 3 weeks) for breast cancer; cyclophosphamide (750 mg/m^2^ intravenously on day 1, every 3 weeks), doxorubicin (50 mg/m^2^ intravenously on day 1, every 3 weeks), vincristine (1.4 mg/m^2^ intravenously on day 1, every 3 weeks, maximum dose: 2 mg/body), and prednisone (40 mg/m^2^ orally on days 1–5, every 3 weeks) with or without rituximab (375 mg/m^2^ intravenously on 1 day before other therapies, every 3 weeks), rituximab (375 mg/m^2^ intravenously on day 1, every 4 weeks) and bendamustine (90 mg/m^2^ intravenously on day 1–2, every 4 weeks), fludarabine (25 mg/m^2^ intravenously on day 2–4, every 4 weeks) and cyclophosphamide (250 mg/m^2^ intravenously on day 2–4, every 4 weeks) with rituximab (375 mg/m^2^ intravenously on day 1, every 4 weeks) for hematologic cancers; and docetaxel (75 mg/m^2^ intravenously on day 1, every 3 weeks) or cabazitaxel (25 mg/m^2^ intravenously on day 1, every 3 weeks) for prostate cancer.

Depending on the patient’s and physician’s preferences, pegfilgrastim 3.6 mg was administered subcutaneously between days 2 and 7. All patients were monitored for adverse events by oncology physicians in accordance with the institution’s guidelines for outpatient chemotherapy. If the patient’s visit was after symptom improvement, they were asked whether they had experienced any intermittent pain in the back, femur, or other anatomic sites. If these episodes of pain were considered bone pain related to pegfilgrastim administration, they were recorded as PIBP. All PIBPs were graded based on the Common Terminology Criteria for Adverse Events version 4.0 (https://evs.nci.nih.gov/ftp1/CTCAE/CTCAE_4.03/Archive/CTCAE_4.0_2009-05-29_QuickReference_8.5x11.pdf). As most patients received multiple cycles of chemotherapy and pegfilgrastim administration, PIBP occurrence was counted per patient if patients had at least one episode of PIBP.

Continuous variables are presented as medians, while categorical variables are presented as counts and percentages. The cutoff value for age was determined using the receiver operating characteristic (ROC) curve for the occurrence of PIBP. Univariate logistic regression analyses were performed to identify risk factors for PIBP, and results are reported as odds ratios (ORs) with 95% confidence interval (CI). Subsequently, we performed multivariate logistic regression analysis to eliminate the effects of confounding factors within items exhibiting statistical significance with *p*-values of < 0.05 in univariate analyses. The target sample size was 300, which was the estimated number of patients who received pegfilgrastim during the study period. All statistical analyses were performed using JMP (version 14.3.0; SAS Institute, Cary, NC, USA).

We identified 315 patients who received pegfilgrastim but excluded 10 patients with incomplete records or unmet criteria. Finally, we analyzed data from 305 eligible patients (median age, 63 years). These patients underwent 1220 chemotherapy cycles with pegfilgrastim as perioperative adjuvant therapy (*n* = 143) or systemic therapy for metastatic or hematologic diseases (*n* = 162). Most patients had a favorable Eastern Cooperative Oncology Group Performance Status (ECOG PS) score of ≤ 1, except for one patient with an ECOG PS score of 2 [10]. Patients receiving antiresorptive agents, such as denosumab or zoledronic acid, were omitted. Opioids or non-steroidal anti-inflammatory drugs (NSAIDs) were regularly used in 13 or 16 patients, respectively (Table 1).

**Table 1:** Patients’ characteristics

Characteristics	N (%)
Sex	
Male	80 (26)
Female	225 (74)
Median age, years (range)	63 (28–87)
**< 55 years**	105 (34)
**≥ 55 years**	200 (66)
Pre-existing osteoporosis	
Yes	8 (3)
No	297 (97)
Type of tumor	
Breast cancer	179 (59)
Hematologic cancer	106 (35)
Prostate cancer	20 (7)
Bone metastasis	
Yes	30 (10)
No	275 (90)
Opioids	
Yes	13 (4)
No	292 (96)
NSAIDs	
Yes	16 (5)
No	289 (95)
Prior chemotherapy	
Yes	28 (9)
No	277 (91)
Type of chemotherapy	
Adjuvant chemotherapy	149 (47)
Chemotherapy alone	162 (53)
Type of prophylaxis	
Primary	251 (82)
Secondary	54 (18)
Pegfilgrastim administration	
Day 2 or 3	129 (42)
Days 4–7	176 (58)

*NSAIDs* Non-steroidal anti-inflammatory drugs

Thirty (10%) patients experienced PIBP of various grades. The first PIBP episode occurred after the first (*n* = 17), second (*n* = 5), or third/subsequent doses (*n* = 8) of pegfilgrastim, with a median post-injection period of 4 (range 1–10) days. The severity of most PIBP cases was grade 1 (*n* = 23), followed by grades 2 (*n* = 5) and 3 (*n* = 2), respectively.

We selected the following items that could affect the incidence of PIBP: sex (male vs. female), age (< 55 years vs. ≥ 55 years), pre-existing osteoporosis, tumor type [solid cancers (breast cancer and prostate cancer) vs. hematologic cancers], bone metastasis (present vs. absent), opioids (yes vs. no), NSAIDs (yes vs. no), history of prior chemotherapy (yes vs. no), chemotherapy type (adjuvant therapy vs. chemotherapy alone), type of prophylaxis for FN (primary vs. secondary), and timing of pegfilgrastim administration (days 2, 3 vs. 4–7) [11]. The threshold of 55 years was determined according to the ROC curve with the area under the curve of 0.744 (*p* < 0.001). We did not select PS because the difference between PS 0 and 1 may not be clinically relevant. Univariate analyses revealed that female sex, younger age (< 55 years), and solid cancers were significantly associated with PIBP (*p* < 0.05). On multivariate logistic regression analysis, only younger age (< 55 years) was independently associated with an increased risk of PIBP (OR 3.62, 95% CI 1.51–8.69, *p* = 0.004) (Table 2).

**Table 2:** Logistic regression analyses of the incidence of pegfilgrastim-induced bone pain

	**PIBP incidence**	**Univariate analysis**			**Multivariate analysis**		
	N (%)	OR	95% CI	*p*-value*	OR	95% CI	*p*-value*
Sex							
Male	2 (3)	Reference			Reference		
Female	28 (12)	5.54	1.29–23.83	0.021	1.909	0.35–10.16	0.455
Age							
< 55 years	21 (20)	5.31	2.32–12.07	> 0.001	3.62	1.51–8.69	0.004
≥ 55 years	9 (5)	Reference			Reference		
Pre-existing osteoporosis							
Yes	0	NA			-		
No	30 (10)	NA			-		
Type of tumor							
Solid cancers	27 (14)	5.39	1.60–18.21	0.007	2.52	0.62–10.19	0.194
Hematologic cancers	3 (3)	Reference			Reference		
Bone metastasis							
Present	3 (10)	0.71	0.21–4.84	0.596	-		
Absent	27 (10)	Reference			-		
Opioids							
Yes	1 (8)	0.79	0.09–6.02	0.784	-		
No	29 (10)	Reference			-		
NSAIDs							
Yes	2 (13)	1.33	0.28–6.16	0.714	-		
No	28 (10)	Reference			-		
Prior chemotherapy							
Yes	4 (14)	1.61	0.52–4.99	0.412	-		
No	26 (9)	Reference			-		
Type of chemotherapy							
Adjuvant chemotherapy	21 (14)	2.93	1.29–6.62	0.010	-		
Chemotherapy alone	9 (6)	Reference			-		
Type of prophylaxis							
Primary	23 (9)	1.48	0.60–3.64	0.410			
Secondary	7 (13)	Reference					
Pegfilgrastim injection							
Days 2 or 3	10 (8)	Reference			-		
Days 4–7	20 (11)	1.53	0.69–3.38	0.298	-		

*NA* Not applicable, *OR* Odds ratio, *CI* Confidence interval, *PIBP* pegfilgrastim-induced bone pain, *NSAIDs* Non-steroidal anti-inflammatory drugs

^*^P-values were calculated using logistic regression analyses

Our findings showed that age < 55 years is a relevant risk factor for PIBP. This could be attributed to the fact that younger patients have greater vital bone marrow functions and possibly lower pain thresholds [12].

Two clinical observational studies have reported a higher risk of PIBP in younger individuals. Moukharskaya et al. evaluated the analgesic efficacy of loratadine prophylaxis in patients who experienced bone pain after pegfilgrastim administration; in the observation stage (*n* = 227), younger patients (≤ 59 years) were more likely to develop significant back or leg bone pain than older patients (> 59 years)(37.0% vs. 23.8%, *p* = 0.039) [8]. Xu et al. performed a meta-analysis of 22 clinical trials, including 1949 patients. They proposed that a history of PIBP and younger age (< 45 years) could be risk factors among patients receiving myelosuppressive chemotherapy with primary prophylaxis using pegfilgrastim [9]. However, the above-mentioned findings had rarely been validated outside the clinical trials before the present study. At first, we applied the cutoff value of 45 years based on the results from Xu et al. [9]. However, to determine the cutoff value in our study more precisely, we replaced the cutoff value of 45 years with 55 years, which was calculated using ROC analysis. Although the patients' background, definition of PIBP, statistical methods, cutoff values for age, and pegfilgrastim doses in these previous studies were different from those in our study, our findings demonstrated that younger age is an established risk factor for PIBP in real-world ontological practice.

A significant limitation of this study is that we could not perform external validation analysis owing to its single-center cohort design. However, our sample size (*n* = 305) can be considered larger than the minimum requirement, which allowed the evaluation of ≤ 3 independent variables in the final model [13]. Another limitation is that a pegfilgrastim dose of 3.6 mg is not commonly administered in most regions outside Japan, as the standard pegfilgrastim dose is 6 mg [7]. In fact, lower pegfilgrastim doses are associated with a lower incidence of PIBP [6]. Therefore, further research is warranted to establish generalizable and reliable risk factors for PIBP.

## Conclusions

Our findings demonstrated that patients aged < 55 years were at a higher risk of PIBP in real-world practice. Accordingly, oncologists should cautiously administer pegfilgrastim and provide careful management for PIBP to these patients.

## Data Availability

Anonymized data from this study are available upon reasonable request to the corresponding author.

## Abbreviations

CI: Confidence interval
C-GSF: Granulocyte-colony stimulating factor
ECOG PS: Eastern Cooperative Oncology Group Performance Status
FN: Febrile neutropenia
OR: Odd’s ratio
PIBP: Pegfilgrastim-induced bone pain
ROC: Receiver operating characteristic
