# Correction to: Higher incidence of pegfilgrastim-induced bone pain in younger patients receiving myelosuppressive chemotherapy: a real-world experience

**DOI:** 10.1186/s40780-023-00284-z

**Published:** 2023-04-14

**Authors:** Shinya Tsuboi, Tatsuya Hayama, Katsuhiro Miura, Akihiro Uchiike, Daisuke Tsutsumi, Takashi Yamauchi, Yoshihiro Hatta, Susumu Ootsuka

**Affiliations:** 1 Department of Pharmacy, Nihon University Itabashi Hospital, 30-1 Oyaguchikamicho, Itabashi city, Tokyo, 173-8610 Japan; 2 Tumor Center, Nihon University Itabashi Hospital, 30-1 Oyaguchikamicho, Itabashi city, Tokyo, 173-8610 Japan; 3 Department of Medicine, Division of Hematology and Rheumatology, Nihon University School of Medicine, 30-1 Oyaguchikamicho, Itabashi city, Tokyo, 173-8610 Japan

**Correction to**: ***J Pharm Health Care Sci*9, 2 (2023)**

10.1186/s40780-022-00272-9

After publication of the original article [1] the authors found an error in the analysis. According to the univariate logistic regression analysis findings summarized in original Table 1, the item “Type of chemotherapy (adjuvant chemotherapy vs. chemotherapy alone)” mentioned odds ratio (OR) of 2.93, with a 95% confidence interval (CI) of 1.29–6.62 and p-value of 0.010.

**Table 1:** Logistic regression analyses for the incidence of pegfilgrastim-induced bone pain

	PIBP incidence	Univariate analysis			Multivariate analysis		
	N (%)	OR	95% CI	*p*-value*	OR	95% CI	*p*-value*
Sex							
Male	2 (3)	Reference			Reference		
Female	28 (12)	5.54	1.29–23.83	0.021	1.99	0.36–11.0	0.413
Age							
<55 years	21 (20)	5.31	2.32–12.07	> 0.001	3.68	1.52–8.90	0.003
≥55 years	9 (5)	Reference			Reference		
Pre-existing osteoporosis							
Yes	0	NA			-		
No	30 (10)	NA			-		
Type of tumor							
Solid cancers	27 (14)	5.39	1.60–18.21	0.007	2.73	0.60–12.43	0.183
Hematologic cancers	3 (3)	Reference			Reference		
Bone metastasis							
Present	3 (10)	0.71	0.21–4.84	0.596	-		
Absent	27 (10)	Reference			-		
Opioids							
Yes	1 (8)	0.79	0.09–6.02	0.784	-		
No	29 (10)	Reference			-		
NSAIDs							
Yes	2 (13)	1.33	0.28–6.16	0.714	-		
No	28 (10)	Reference			-		
Prior chemotherapy							
Yes	4 (14)	1.61	0.52–4.99	0.412	-		
No	26 (9)	Reference			-		
Type of chemotherapy							
Adjuvant chemotherapy	21 (14)	2.93	1.29–6.62	0.010	1.14	0.40–3.23	0.804
Chemotherapy alone	9 (6)	Reference			Reference		
Type of prophylaxis							
Primary	23 (9)	1.48	0.60–3.64	0.410			
Secondary	7 (13)	Reference					
Pegfilgrastim injection							
Day 2 or 3	10 (8)	Reference			-		
Days 4–7	20 (11)	1.53	0.69–3.38	0.298	-		

NA, not applicable; OR, odds ratio; CI, confidence interval; PIBP, pegfilgrastim-induced bone pain; NSAIDs, non-steroidal anti-inflammatory drugs.

* *P*-values were calculated by logistic regression analyses.

According to the statistical method used, this item should be included in multivariate analysis. However, the results for this clinical factor in the multivariate analysis were blank. The correct version of Table 1 is included in this correction article.

Accordingly, the corrected OR for younger age (< 55 years) related to pegfilgrastim-induced bone pain is 3.68 (95% CI 1.52–8.90, p = 0.003). Owing to the minimum alterations in OR and p-values in multivariate analysis, the authors believe that this correction does not significantly impact the study results.
